# Modulation of immunity in young-adult and aged squirrel, *Funambulus pennanti *by melatonin and p-chlorophenylalanine

**DOI:** 10.1186/1742-4933-6-5

**Published:** 2009-04-23

**Authors:** Seema Rai, Chandana Haldar, Rajesh Singh

**Affiliations:** 1 Department of Zoology, Pineal Research Lab, Banaras Hindu University, Varanasi – 221005, India

## Abstract

**Background:**

Our interest was to find out whether pineal gland and their by melatonin act as modulator of immunosenescence. Parachlorophenylalanine (PCPA) – a β adrenergic blocker, is known to perform chemical pinealectomy (Px) by suppressing indirectly the substrate 5-hydroxytryptamine (5-HT) for melatonin synthesis and thereby melatonin itself. The role of PCPA, alone and in combination with melatonin was recorded in immunomodulation and free radical load in spleen of young adult and aged seasonal breeder Indian palm squirrel *Funambulus pennanti*.

**Results:**

Aged squirrel presented reduced immune parameters (i.e. total leukocyte count (TLC), Lymphocytes Count (LC), % stimulation ratio of splenocytes (% SR) against T cell mitogen concanavalin A (Con A), delayed type hypersensitivity (DTH) to oxazolone) when compared to young adult group. Melatonin administration (25 μg/100 g body mass/day) significantly increased the immune parameters in aged more than the young adult squirrel while PCPA administration (4.5 mg/100 g body mass/day) reduced all the immune parameters more significantly in young than aged. Combination of PCPA and melatonin significantly increased the immune parameters to the normal control level of both the age groups. TBARS level was significantly high in aged than the young group. PCPA treatment increased TBARS level of young and aged squirrels both while melatonin treatment decreased it even than the controls. Nighttime peripheral melatonin level was low in control aged group than the young group. Melatonin injection at evening hours significantly increased the peripheral level of nighttime melatonin, while combined injection of PCPA and melatonin brought it to control level in both aged and young adult squirrels.

**Conclusion:**

PCPA suppressed immune status more in aged than in adult by reducing melatonin level as it did chemical Px. Melatonin level decreased in control aged squirrels and so there was a decrease in immune parameters with a concomitant increase in free radical load of spleen. Decreased immune status can be restored following melatonin injection which decreased free radical load of spleen and suggest that immune organs of aged squirrels were sensitive to melatonin. Increased free radical load and decreased peripheral melatonin could be one of the reasons of immunosenescence.

## Background

Melatonin (N-acetyl-5-methoxytryptamine) is synthesized mainly in pineal gland. It plays a major role in regulating sexual maturity, reproductive cycle, stress and the immune responses [1-3]. On the other hand, it has also been observed that melatonin declines with age [4] and may act as a key regulator in ageing and senescence [5,6]. Aging is associated with a decline in immune function known as immunosenescence. This situation implies increased susceptibility to infectious disease due to decreased capacity of the immune system to respond to antigenic stimulation [7]. Many hormones also decline with advancing age (growth hormone (GH), estrogen, dehydroepiandrosterone and melatonin) playing a significant role in immunosenescence [8]. Among those, melatonin has been demonstrated to bear a general immune-enhancing effect in many animal species including humans [9]. Whether this immune- enhancing property of melatonin exists in aged animals needs to be verified. Pharmacological and surgical pinealectomy also modulate various immune parameters including plaque-forming cells and blastogenic responses of splenocytes and thymocytes to various mitogens [10]. Further, disruption of the night – time peak of melatonin completely abolished Colony Forming Unit- Granulocyte Macrophages (CFU-GM) proliferation of bone marrow cells [11,12].

In general PCPA- a â adrenergic blocker, is known as serotonin depletor, also had several behavioral effects such as induction of aggression in pregnant females and the sexual excitement in males. Initially, Marco et al, (1982) [13] suggested a double effect of PCPA on LH, FSH, prolactin and also on ovarian cycle including estrogen secretion. In later years handful of report suggested the effects of less serotonin due to PCPA on reproductive behavior [14,15], increased maternal aggression [16] and swimming behavior of young rats [17].

PCPA has been proposed as an indirect antagonist of melatonin as it inhibits the melatonin biosynthesis pathway by acting on tryptophane hydroxylase the enzyme responsible for synthesis of serotonin (serotonin is the substrate for melatonin). PCPA, being a specific depletor of serotonin (5-HT) contributes also to the prenatal development of the central nervous system, acting as a morphogen in the young embryo and later as a neurotransmitter [18,19]. If endogenous melatonin synthesis is blocked by administration of propanolol (PRO) or p-chlorophenylalanine (PCPA) during evening hrs, it, significantly depresses the antibody production in human and mice [20,21].

Further, the basic problem of aging is the declined efficiency in all organisms. This will lead to dysfunction and degeneration, increase in free radical load and reactive oxygen species (ROS) in particular. Further, direct free radical scavenging [22] and indirect antioxidant stimulatory property of melatonin was used in number of studies for improving metabolic function [23,24]. Hardly any report exist explaining the age related changes in immune function and free radical load along with protective effect of melatonin on them in any wild mammals. Therefore, in the present study we recorded the immune status and free radical load of aged and compared it with young adult male squirrel, *Funambulus pennanti *on one hand and effect of melatonin, PCPA (a specific depletor of serotonin level) and PCPA plus melatonin on the improvement of immune functions on the other. We selected Indian palm squirrel, *Funambulus pennanti *as animal model because most of the parameters and doses for melatonin and PCPA related to present study has already been established which eventually helped in planning this study.

## Materials and methods

All the experiments on the animals were conducted in accordance with Institutional practice and with the framework of revised Animals (Specific Procedure) Act of 2002 of Govt. of India on animal welfare.

### Animal Maintenance

Squirrels were collected from the vicinity of Varanasi (Latitude 25°, 18 ' N, Longitude 83°, 01' E) as they were available in plenty of numbers and were maintained in laboratory under ambient conditions adapted for several weeks for young adult squirrels and years for aged squirrels. Young adults (weighing 70 ± 10 g, ~5 months old) and aged (weighing 140 ± 10 g, ~2.0 years old) squirrels as judged by incisor length and cranium diameter; [25] were used for present study. The animals were maintained in an open air fenced area, where they can move freely. During experiment they were housed in wire net cages (25" × 25" × 30" in size) in the same housing area. The squirrels were fed with soaked gram, *Cicer arietinum *along with other seasonal food grains, nuts and water *ad libitum*. Squirrels were then randomized and divided into following groups containing 12 squirrels as mentioned in Table 1.

**Table 1:** 

Groups	Treatment	No. of young/aged squirrels
Control	0.9% saline/day	12/12

PCPA treated	4.5 mg/squirrel/day	12/12

PCPA + Melatonin	4.5 mg/squirrel/day +25 μg/squirrel/day	12/12

Melatonin	25 μg/squirrel/day	12/12

### Drug and treatment protocols

Para-chlorophenylalanine (PCPA) and melatonin were purchased from (SIGMA, St. Louis, Missouri, USA). The control squirrels were injected (subcutaneously) with the normal ethanolic (20 μl) saline (0.9%NaCl) 0.1 ml/day. Melatonin solution was made by dissolving it in few drops (20 μl) of ethanol and then diluted with normal saline (0.9% NaCl) up to the desired concentration. PCPA was dissolved in 1 N NaOH and diluted with PBS. Melatonin 25 μg/squirrel/day (subcutaneously; S.C.) and PCPA, 4.5 mg/squirrel/day (intra peritoneal; i. p.) at evening hrs.(4.30–5.30 pm, i.e.1.30 hrs before lights off) were injected daily for 60 consecutive days.

At the end of the experiment (60^th ^days), six animals from each group of young adult and aged were subjected for the Delayed Type Hypersensitivity (DTH) response test for ear swelling tests according to Phanuphak et al, (1974) and Disis et al, (2000) [26,27]. Remaining six squirrels following their sacrifice at evening hrs (5.00 – 6.00 PM) after complete anesthesia (anesthetic ether) were subjected for spleen weight analysis, MDA assay [28] and blastogenic response of splenocytes. Blood plasma was separated by centrifugation and was stored at -20° for radioimmunoassay (RIA) of melatonin. Blastogenic response of splenocytes was noted in terms of tritiated thymidine uptake (^3^H-TdR; Specific activity 8.9 ci mM; BARC Mumbai, India) against T cell mitogen concanavalin A [29] and expressed in terms of percent stimulation ratio (% SR) of splenocytes.

### RIA of Melatonin

Melatonin RIA was done following modified method of Rollag and Niswender (1976) [30] practiced in our lab by Haldar et al, (2006) [31], using Stock Grand anti-melatonin antibody (Stock Grand, Surrey, UK). The recovery, accuracy and sensitivity for the melatonin RIA were 92%, 0.98 and 10 pg/ml respectively. Intra and Inter-assay variation of melatonin were 9.0% and 15% respectively.

### Splenocyte culture *in vitro*

Spleen was dissected out and cleaned from adhered fatty tissues and immediately, placed in chilled PBS. Spleen was then minced, passed through a steel screen of 400 meshes, collected into sterile centrifuge tube and washed twice with RPMI-1640. The cell viability was checked by trypan blue exclusion method (average viability was noted 99%). The erythrocytes in spleen cell suspension were lysed with cold 0.5% Tris buffer and 0.84% NH_4_Cl mixed in 1:10 ratio and adjusted to pH 7.2. The cell suspension was adjusted to 1 × 10^6 ^cells/ml in RPMI 1640, containing sodium bicarbonate, antibiotics (Penicillin 100 IU/ml, streptomycin 100 μg/ml, Gentamycin 100 μg/ml) and 10% fetal calf serum (Sigma, USA).

### Blastogenic response of splenocytes to mitogen

The blastogenic response to 4.5 μg/ml of the mitogen Con A was evaluated following the method of Pauley and Sokal, 1972 [29]. The mononuclear lymphoid cells (1 × 10^6 ^cells/ml) were incubated with medium in a plastic 96-well tissue culture plate for 72 hr. The lymphoid cell proliferation was assayed by pulse labeling with tritiated thymidine (^3^H-TdR; Specific activity 8.9 ci mM; BARC Mumbai, India), 18 hr before the end of incubation period. A 0.1 ml aliquot was counted using a liquid scintillation counter (Packard, USA). Results are expressed as ^3^H-TdR incorporation in counts per minute as follows:



### Lipid peroxidation (LPO) assay

Free radicals have a very short half-life, which makes them very hard to measure in the laboratory. A commonly used alternate approach measures markers of free radicals rather than the actual radical. Thiobarbituric acid reactive substances (TBARS) are produced during oxidative damage to cell membrane. Malonaldehyde (MDA), one of the major lipid breakdown product and commonly used parameter will be assessed by the method of Ohkawa et al, 1978 [28].

All spleens were excised and weighed for the preparation of 10% tissue homogenates in 20 mM Tris Hydrochloride (HCl) buffer (pH 7.4). The homogenates were centrifuged at 3000 *g *for 15 min at 4°C and supernatant was subjected to thiobarbituric acid (TBA) assay by mixing it with 8.1% SDS, 20% acetic acid, 0.8% TBA and boiling for 1 h at 95°C. The reaction mixture was immediately cooled in running water and vigorously shaken with n-butanol and pyridine reagent (15:1) and centrifuged for 10 min at 1500 *g*. The absorbance of the upper phase was measured at 534 nm. LPO was expressed as TBARS in nmol/g tissue wt., by taking 1,1,3,3 tetraethoxy propane (TEP) as standard. The standard curve was calibrated using 10 nM concentration of TEP.

### Statistics

All the data were expressed as means ± SEM of at least 6 animals per point. Data comparisons were statistically analyzed by using ANOVA followed by Student Newman-Keuls' multiple range tests among the groups while, student t test was performed to compare the aged group with young one. The differences were considered statistically significant when *p *< 0.05.

## Results

### Spleen weight

We compared our data first between the two groups (aged and young) and then among aged and young adult groups following treatment with in the groups. More than fifty percent decrease in spleen weight of aged squirrel was recorded when compared with young group.

**Figure 1:**
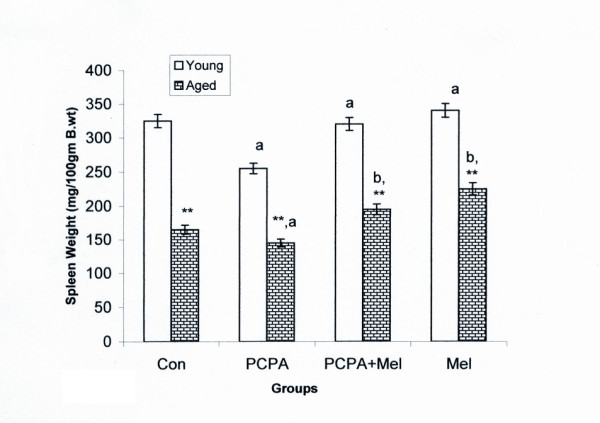
**Effect of p-chlorophenylalanine and melatonin treatment on spleen weight of young adult and aged squirrels**. Histogram represents Mean ± SE; n = 12 for each group. Con = Control, PCPA = para-chlorophenylalanine, PCPA + Mel = para-chlorophenylalanine + Melatonin, Mel = Melatonin Significance of difference aged vs young adult; * = P < 0.05; ** = P, < 0.01 Significance of difference treated aged vs treated young adult; a = P < 0.05; b = P, < 0.01.

PCPA treatment decreased spleen weight of aged and young both groups of squirrels when compared with saline treated control group. Further, a combined treatment of melatonin along with PCPA significantly (P < 0.01) increased the spleen weight of aged squirrels only without affecting the spleen weight of young adult squirrel when compared with their respective controls. Moreover, melatonin alone given to the aged squirrels showed a significant (P < 0.01) increase in spleen weight when compared with control, PCPA and PCPA plus melatonin treated groups (Figure 1).

### Total leukocyte and lymphocyte count (TLC and LC)

**Figure 2:**
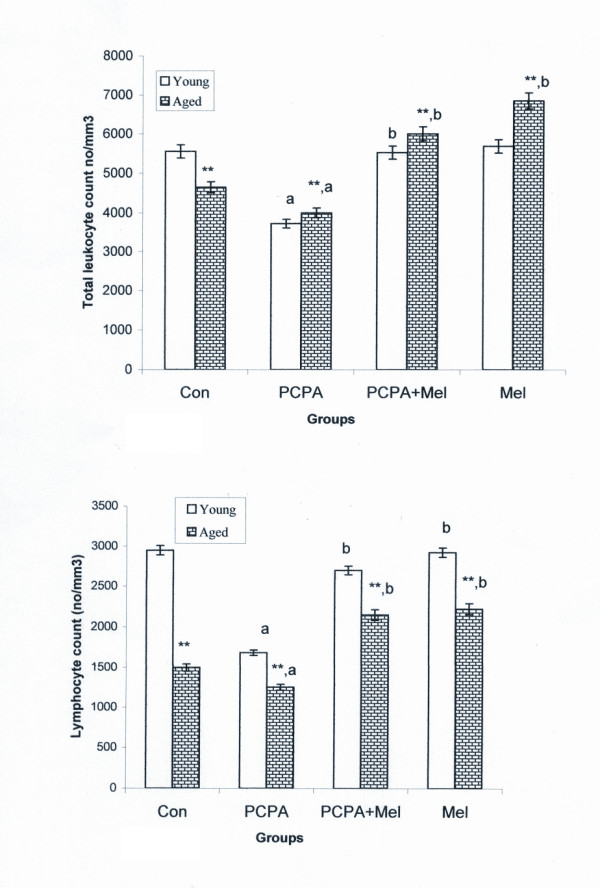
**Effect of p-chlorophenylalanine and melatonin treatment on total leukocyte (TLC; Figure 2a.) and lymphocyte count (LC; Figure. 2b) of young adult and aged squirrels**. Histogram represents Mean ± SE; n = 12 for each group. Con = Control, PCPA = para-chlorophenylalanine, PCPA + Mel = para-chlorophenylalanine + Melatonin, Mel = Melatonin Significance of difference aged vs young adult; * = P < 0.05; ** = P, < 0.01 Significance of difference treated aged vs treated young adult; a = P < 0.05; b = P, < 0.01.

There was a decrease in no of TLC and LC in control aged group when compared with control young adult group. PCPA treatment significantly (P < 0.01) decreased TLC and LC of young adult and aged squirrels when compared with their respective control. Further, melatonin and PCPA treatment to young adult and aged squirrels significantly (P < 0.01) enhanced the TLC and LC when compared with the control and PCPA treated groups. Melatonin alone to the aged squirrels significantly (p < 0.01) increased TLC and LC when compared with the control and PCPA treated aged squirrels and had similar value as the combined treatment of PCPA plus melatonin (Figure 2a, b). Melatonin alone or in combination with PCPA had no significant effect on TLC and LC of young adult group when compared with young adult control group.

### Delayed Type Hypersensitivity (DTH) response to oxazolone

**Figure 3:**
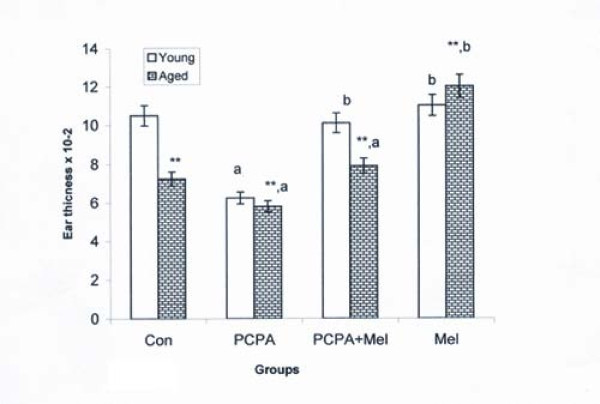
**Effect of p-chlorophenylalanine and melatonin treatment on Delayed Type Hypersensitivity (DTH) response to oxazolone (in terms of ear thickness) of young adult and aged squirrels**. Histogram represents Mean ± SE; n = 12 for each group. Con = Control, PCPA = para-chlorophenylalanine, PCPA + Mel = para-chlorophenylalanine + Melatonin, Mel = Melatonin. Significance of difference aged vs young adult; * = P < 0.05; ** = P, < 0.01. Significance of difference treated aged vs treated young adult; a = P < 0.05; b = P, < 0.01.

Aged squirrels presented a general decline in DTH response than the young adult squirrels. A significant decrease of DTH response was noted following the PCPA treatment in young squirrel when compared with the control group while this decline was less evident in aged group. Combined treatment of melatonin and PCPA had no effect on DTH response of young adult but aged squirrels showed significant increased when compared with their respective saline treated control group of squirrels. Melatonin treatment alone increased significantly (P < 0.01) DTH response in both aged but not in young squirrels when compared with saline treated control group (Figure 3).

### Blastogenic response of splenocytes in terms of percent stimulation ratio (% SR)

**Figure 4:**
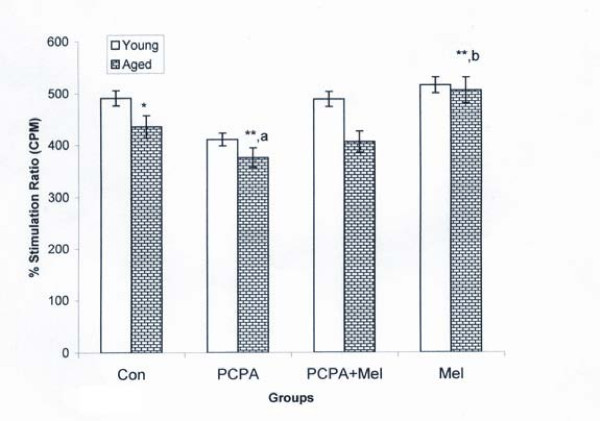
**Effect of para-chlorophenylalanine and melatonin treatment on blastogenic response against T cell mitogen Concanavalin (Con A) in terms of percent stimulation ratio (%SR) of young adult and aged squirrels**. Histogram represents Mean Mean ± SE; n = 12 for each group. Con = Control, PCPA = para-chlorophenylalanine, PCPA + Mel = para-chlorophenylalanine + Melatonin, Mel = Melatonin. Significance of difference aged vs young adult; * = P < 0.05; ** = P, < 0.01. Significance of difference treated aged vs treated young adult; a = P < 0.05; b = P, < 0.01.

Control aged squirrel had significantly less %SR than young adult. PCPA treatment significantly (P < 0.01) decreased % SR of splenocytes in young adult and aged squirrels when compared with saline treated control group. Further, combined treatment of PCPA and melatonin to young adult and aged squirrels had no recovery effect on % SR of splenocytes when compared with saline treated control group. In addition melatonin treatment alone to the young adult squirrel could not show any effect while a significant (P < 0.01) increase was noted in % SR of splenocytes of aged squirrel when compared with their respective saline treated control, PCPA and PCPA plus melatonin treated groups (Figure 4).

### Radioimmunoassay of Melatonin

**Figure 5:**
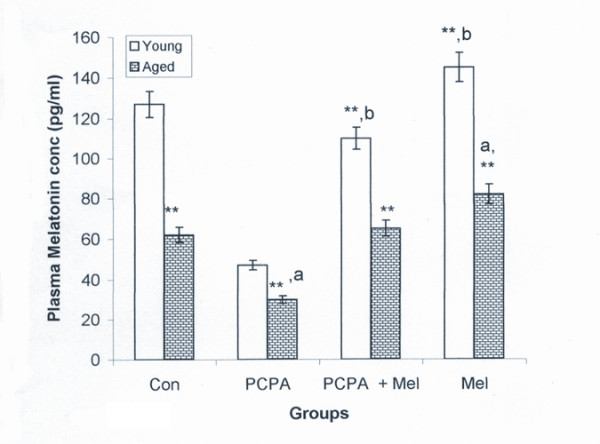
**Effect of para-chlorophenylalanine and melatonin treatment on endogenous level of melatonin of young adult and aged squirrel**. Histogram represents Mean ± SE; n = 12 for each group. Con = Control, PCPA = para-chlorophenylalanine, PCPA + Mel = para-chlorophenylalanine + Melatonin, Mel = Melatonin. Significance of difference aged vs young adult; * = P < 0.05; ** = P < 0.01. Significance of difference treated aged vs treated young adult; a = P < 0.05; b = P < 0.01.

Peripheral melatonin level was significantly less in aged group of squirrel than young adult group. Significant (P < 0.01) decrease in plasma melatonin was noted in young as well as aged squirrels following PCPA treatment when compared with their saline treated control group. Combined treatment of PCPA and melatonin in aged squirrels had no effect on melatonin level but it decreased melatonin level in young adult squirrels when compared with saline treated control group. However, melatonin treatment alone significantly increased its level in aged and young adult squirrels (Figure 5).

### TBARS level

**Figure 6:**
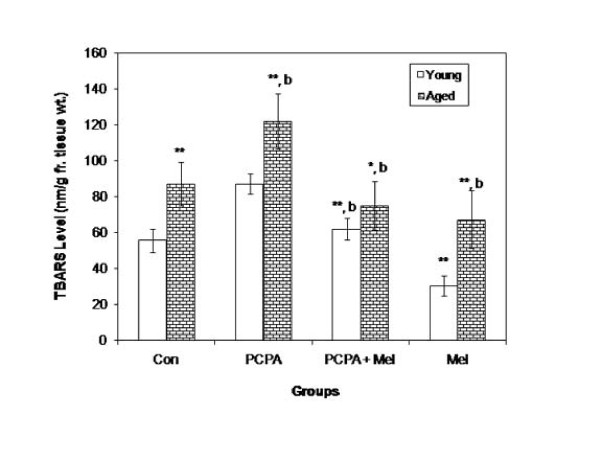
**Effect of para-chlorophenylalanine and melatonin treatment on TBARS level of melatonin of young adult and aged squirrels**. Histogram represents Mean ± SE; n = 12 for each group. Con = Control, PCPA = para-chlorophenylalanine, PCPA + Mel = para-chlorophenylalanine + Melatonin, Mel = Melatonin. Significance of difference aged vs young adult; * = P < 0.05; ** = P < 0.01; Significance of difference treated aged vs treated young adult; a = P < 0.05; b = P, < 0.01.

Significant increase was noted in TBARS level of aged squirrels as compared with the young group. PCPA treatment caused significant increase in lipid peroxidation in both the age groups while melatonin in combination with PCPA reduced it to the level of control group. However, injection of melatonin alone significantly decreased the TBARS level in young as well as in aged squirrels when compared with saline treated control group (Figure 6).

## Discussion

Researchers are actively engaged in solving the age related decline in various physiological functions. Evidences supporting the properties of melatonin that affect the ageing process comes from the studies in mice where pathological changes resembling senescence occurred when pineal gland was destroyed. When pineal gland of young mice was grafted in older animals they exhibited a prolonged life span [32,33]. We have already proposed that melatonin act as an immunostimulator in *Funambulus pennanti *when tested in *in vivo *as well as *in vitro *[8]. Whether the immunostimulatory effect of melatonin is age dependent in this seasonal breeder, *F. pennanti *is being reported here in order to specify the role of melatonin in immunosenescence. We used an indirect blocker of melatonin synthesis i.e. PCPA to support it. Our data clearly suggest an age related decline of the immune system (almost all the parameters) associated with the decrease in plasma melatonin level [34]. Further, a significant suppression of spleen weight following treatment of PCPA in young squirrels was noted while, aged squirrels showed no such reduction as they had already reduced spleen weight when compared with the young adult squirrels. Combined treatment of melatonin and PCPA showed a significant increase in splenic mass when compared with control and PCPA treated group. This indicates that melatonin is able to compromise the PCPA induced suppression of spleen mass and thereby immunity. The magnitudes of the PCPA effect in reducing immune activity were more prominent in young squirrels than the aged ones. Our above observation support the earlier report of Maestroni and his coworkers (1986) [35] that deprivation of nocturnal circulating melatonin by constant light exposure (physiological pinealectomy) and administration of propanolol or PCPA (chemical pinealectomy) reduced the splenic and thymic cellularity and that pinealectomy caused thymic involution [36] pointing towards the significance of the melatonin in the maintenance of the lymphoid organ activity. This could be the reason behind observation when we noted that PCPA significantly suppressed the total leukocyte and lymphocyte count in young adult squirrels, while no suppression was noted in the aged squirrels as their TLC and LC number were already quite low. However, melatonin treatment to the aged squirrel increased the peripheral TLC and LC once more supporting the immunostimulatry action of it which is independent of age.

The Delayed Type Hypersensitivity (DTH) response to oxazolone as a measure of T-cell mediated immune function [26,27] and splenocytes proliferative response to the mitogen Con A which reflected that PCPA treatment significantly suppressed the cell mediated immunity and immune status while melatonin treatment improved the suppressed immune functions, but the magnitude of the response was more prominent in aged squirrels than the young squirrels. Decrease in plasma melatonin level following PCPA signifies its action as chemical pinealectomy in young adult more prominently.

The present result of increased lipid peroxidation (LPO) production in aged squirrels suggest involvement of free radicals and the role of redox imbalance could be a result of immunosenescence which was restored following the melatonin treatment. The data of the present study is also in support of the previous literatures suggesting the role of PCPA on increased lipid peroxidation products and reduced glutathione content in renal and brain tissue [37]. Our results also support the additive pro-oxidant action of PCPA in spleen to exacerbate the oxidative stress during ageing when the LPO was noted very high which might have reduced immune functions as well as proved the possible antioxidant property of melatonin. Melatonin stimulates the production of glutathione [38] and therefore immunoenhancing role of melatonin following its administration may be partly due to its influence on the maintenance of intracellular glutathione level.

PCPA treatment was more effective in reducing the immune parameter via reducing melatonin level in young group, whereas in aged squirrels it did not suppress the immune parameters significantly because of (i) low level of melatonin and (ii) already low level of immunity. This is the reason that melatonin treatment in young squirrels could not affect the studied immune parameters whereas in aged squirrels it was more effective in inducing the immune status.

## Conclusion

The result reported here indicates that the immunemodulatory role of melatonin is age independent and the immunosenescence is due to low level of melatonin production with age. The mechanism by which melatonin exerts its immuno-potentiating action could be partially explained via its action in reducing the free radical load [39].

## Competing interests

The authors declare that they have no competing interests.

## Authors' contributions

RS participated in execution of the experiments.  SR was involved in estimations (RIA and Free radicals etc.), statistical analysis, drafting of manuscript etc. CH made contribution in conception of experimental project, planning, interpretation of data critical evaluation of MS for intellectual content and has given final touch in preparation of MS for publication.
